# Perspectives on using decision-making nudges in physician-patient communications

**DOI:** 10.1371/journal.pone.0202874

**Published:** 2018-09-19

**Authors:** Ilona Fridman, Joanna L. Hart, Kuldeep N. Yadav, E. Tory Higgins

**Affiliations:** 1 Columbia Business School, Columbia University, New York, NY, United States of America; 2 Palliative and Advanced Illness Research (PAIR) Center, University of Pennsylvania, Philadelphia, PA, United States of America; 3 Fostering Improvement in End-of-Life Decision Science (FIELDS) Program, University of Pennsylvania, Philadelphia, PA, United States of America; 4 Center of Health Incentives and Behavioral Economics (CHIBE), University of Pennsylvania, Philadelphia, PA, United States of America; 5 Leonard Davis Institute of Health Economics, University of Pennsylvania, Philadelphia, PA, United States of America; 6 Division of Pulmonary, Allergy, and Critical Care Medicine, University of Pennsylvania, Philadelphia, PA, United States of America; 7 Department of Medical Ethics and Health Policy, University of Pennsylvania, Philadelphia, PA, United States of America; 8 Department of Psychology, Columbia University, New York, NY, United States of America; Public Library of Science, UNITED KINGDOM

## Abstract

Patients engaging in shared decision making must weigh the likelihood of positive and negative outcomes and deal with uncertainty and negative emotions in the situations where desirable options might not be available. The use of “nudges,” or communication techniques that influence patients’ choices in a predictable direction, may assist patients in making complex decisions. However, nudging patients may be perceived as inappropriate influence on patients’ choices. We sought to determine whether key stakeholders, physicians, and laypersons without clinical training consider the use of nudges to be ethical and appropriate in medical decision making. Eighty-nine resident-physicians and 336 Mechanical-Turk workers (i.e., non-clinicians) evaluated two hypothetical preference-sensitive situations, in which a patient with advanced cancer chooses between chemotherapy and hospice care. We varied the following: (1) whether or not the patient’s decision was influenced by a mistaken judgment (i.e., decision-making bias) and (2) whether or not the physician used a nudge. Each participant reported the extent to which the communication was ethical, appropriate, and desirable. Both physicians and non-clinicians considered using nudges more positively than not using them, regardless of an initial decision-making bias in patients’ considerations. Decomposing this effect, we found that physicians viewed the nudge that endorsed hospice care more favorably than the nudge that endorsed chemotherapy, while non-clinicians viewed the nudge that endorsed chemotherapy more favorably than the nudge that endorsed hospice care. We discuss implications and propose exploring further physicians’ and patients’ differences in the perception of nudges; the differences may suggest limitations for using nudges in medical decisions.

## Introduction

Patients engaging in shared decision making often struggle to match available treatment options with their own goals and preferences. These decisions may be influenced by predictable cognitive errors that negatively impact patient well-being. Research in behavior economics has demonstrated that, while making decisions, individuals can assign different weights to losses and corresponding gains, imperfectly predict future preferences, and inaccurately remember their past experiences [1]. Providing relevant information might not be enough to prevent harmful decisions. For example, studies have demonstrated that calorie labeling was not effective in reducing calories intake [2]. To assist patients in navigating decision-making processes and counteract cognitive errors, physicians may be able to use “nudges,” or communication strategies with predictable effects on the resulting patient choice, [3].

Nudges are the aspects of choice architecture or a communication that alters behavior in a predictable way without changing the outcomes of available options or restricting individual’s ability to choose [4]. A well-explored nudge is setting default options. Default enrollment means that individuals are automatically included in a program. At the same time, they may quit the program easily if they want. For example, in some DMV offices in the U.S., individuals are enrolled in an organ donation program automatically when they receive a driving license. To ensure individuals’ freedom of choice, DMV officers inform everyone that they are free to dis-enroll from the program. This and other default nudges substantially increase participation in various programs [5–9]. However, given the influential power of nudges, physicians’ use of such communication strategies that influence patients’ choice in a predictable way may be evaluated as unethical persuasion [10–13]. Specifically, some might consider nudges as adversely influencing patients’ ability to choose according to their own preferences.

Researchers argue that nudges relating to preventive medicine might be acceptable because it is often clear which option has a positive outcome for patients. Nonetheless, the use of nudges in preference-sensitive decisions, such as end-of-life care, raises strong ethical concerns and requires empirical investigation [14]. Evidence of how key stakeholders, such as patients and physicians, view physicians’ use of nudges is limited. In this study, we aim to investigate how non-clinicians (i.e., potential patients) and physicians perceive the use of nudges in preference-sensitive, end-of-life decisions.

Additionally, nudges often are considered as a tool to correct individuals’ biases [15, 16]. It might be that, when biases (i.e., cognitive error) are present in patients’ judgments, physicians and non-clinicians would consider nudging to be more ethical, appropriate and desirable. To explore this possibility, we randomly informed some participants that a patient had a bias in the initial judgment. We expected that participants who were informed about the bias might perceive nudges more positively than these who were not informed about the bias.

## Methods

We conducted an experimental study among non-clinicians and resident-physicians. Non-clinicians were reached via a Mechanical Turk (Mturk) online panel in July 2016. Mturk is an online labor market in which participants produce psychometrical data [17, 18] . Recent studies have shown that up to 85% Mturk workers produce valid responses [19].

Physicians in their final year of residency at a single health care system were identified through the websites and staff directories. They received a link to the electronic survey via their professional emails, followed by three reminders at the most, from September 2016 to November 2016. Both Mturk participants and resident physicians received compensation for their participation. The study was approved by the University of Pennsylvania Institutional Review Board. Participants were 18 years of age or older. A written form of informed consent was waived by IRB. Participants instead read an informational sheet online before entering the survey. They were informed about study procedures, confidentiality of individual responses and that their participation was voluntary. After reading the information about the study, participants were asked if they agree to take part in the survey. These who agreed were allowed to proceed to the survey.

Designed in Qualtrics, the survey consisted of two decision-making vignettes describing patients deciding between chemotherapy and hospice care for incurable cancer. Two attributes of each vignette varied randomly among participants, see Table 1: (1) whether or not the patient initially made a choice based on a mistaken judgment (decision-making bias) and (2) whether or not the physician used a decision-making nudge (e.g., information framing and social comparisons) when communicating with the patient (for details see Table 1). Both vignettes stated explicitly that if the physician uses a decision-making nudge it will increase the likelihood that the patient would follow the advice. After each vignette, participants evaluated the ethics of the communications in several dimensions including, desirability, appropriateness, patients’ autonomy and others, described below. Both vignettes and dependent variables are reported verbatim in supplementary materials (see S1 and S2 Tables)

**Table 1:** Decision-making vignettes and manipulations.

	Decision-making vignette 1: Hospice endorsed	Decision-making vignette 2: Chemotherapy endorsed
Patient’s initial treatment choice	Chemotherapy	Hospice care
Patient’s bias (manipulation 1) *	Overestimating positive outcomes: the patient mistakenly believes that he may live much longer or be cured of the cancer	Availability bias: the patients’ friend told her that when chemotherapy passes through veins, it causes terrible pain
Physician’s rationale	Chemotherapy is not curative and will do more harm than good for him. It will cause tiredness and pain and increase the risk of infection.	Chemotherapy would likely benefit the patient by prolonging her life up to 18–24 months.
Physician’s recommendation	Hospice care	Chemotherapy
Decision-making nudge	Framing: if the patient is focused on avoiding losing a chance for a cure by choosing chemotherapy, the physician could re-focus the patient's attention. To do so, he should mention gains the patient could achieve by choosing comfort care instead of chemotherapy (i.e. more meaningful time with his family and friends). Prior research suggests that this decision-making approach will make it more likely that the patient will choose comfort-oriented care for advanced cancer.	Social comparison: The physician could inform the patient that a recent patient in a similar situation received the same treatment. It shrunk her cancer and allowed her to live longer than she would have been expected to live without any treatment. Additionally, medications helped manage the side effects of chemotherapy. Prior research has shown that this approach will make it more likely that the patient will choose chemotherapy.
Physician decision about using nudge (manipulation 2)	The physician decides to **use/not to use** this decision-making approach to influence the patient's choice.	The physician decides to **use/not to use** this decision-making approach to influence the patient's choice.

* randomized: presented only for a half of the participants

### Materials and measures

#### Nudges

We chose to explore the nudges that influence individuals’ without their conscious awareness about the nudge or influence. These nudges are considered to be more controversial than nudges that appeal to individuals’ deliberative thinking [20]. In the first vignette, we used an information framing to nudge a person to reconsider his initial opinion [3]. In the second vignette, we used social comparison to nudge the patient to agree for chemotherapy [21]. In both vignettes, if patients experienced a nudge, they were neither informed nor aware that the nudge would increase the probability that they would agree with physicians’ advice.

#### Decision-making bias

In Vignette 1, for those participants who we informed about the patient’s bias, we stated that patients’ judgments are based on an optimistic bias, which is patients’ tendency to be overly optimistic about treatments’ outcomes [22]. Participants were randomly assigned either to a “no information” condition (no bias condition) or to read the following (informed bias condition): “overestimating the power of chemotherapy, the patient fully believes that chemotherapy will help him live longer and even cure his cancer.” In Vignette 2, we utilized availability bias that is patients’ tendency to base their judgments on one single event in the past [11, 23]. Participants were randomly assigned either to no information (no bias condition) or to read the following (informed bias condition): “During the consultation, she [patient] tells her physician that she did not choose chemotherapy because she is afraid of its “horrible” side effects. She explains that her friend told her that chemotherapy causes terrible pain and a burning sensation in the blood vessels”.

#### Evaluation of communications

Participants’ evaluation of communications included several components, reported in Table 2. Participants rated their agreement with each statement on a 7-point Likert scale (1 = strongly disagree, 7 = strongly agree). The first component included ethics of communications. It consisted of a statement: “the physician communication was ethical.” The second component measured the acceptability of communications (reversed), “the physician communication was unacceptable (reversed).” The desirability component was measured with the following question for non-clinicians, “I would prefer that my physician acts this way,” and with the following question for clinicians, “I would use this approach in a similar situation” Ethics, acceptability and desirability components were adopted from a prior study that evaluated ethics of clinical communications [24].

**Table 2:** Dependent variables.

Variable	Item	Cronbach’ alfa non-clinicians Vignette1/Vignette 2	Cronbach’ alfa Physicians Vignette1/Vignette 2
Ethics	The physician's communication was ethical.	—	—
Desirability	I would prefer my physician to use this approach in a similar situation (non-clinicians) I would use the same approach (Physicians)	—	—
Acceptability	The physician's behavior was unacceptable. (Reversed)	—	—
Effectiveness	The physician did the best she/he could for this patient. The physician should have tried harder to help the patient make a decision (reversed)	.74 /81	.62/75
Autonomy	The physician respected the autonomy (freedom of choice) of the patient in this communication. The physician acted unacceptably paternalistic (by overriding patients’ preferences). (reversed)	.65/.57	.65/.68
Benevolence	The physician acted in the best interests of the patient.	—	—
Respect to Patient	The physician treated the patient with dignity and respect	—	—
Trust	The patient will trust in his/her physician after this communication.	—	—

The effectiveness of nudges has been shown to be an important aspect in how individuals evaluate behavioral nudges [25]. Thus, another component in the study assessed the effectiveness of communications, with the following two statements: “The physician did the best she/he could for this patient,” and “The physician should have tried harder to help the patient make a decision (reversed)”.

Participants also rated to what extent the patient in the vignette would trust his/her physician after the described communication. This component was included following the work of Tannenbaum [26], who demonstrated that trust in the source of a nudge influences how individuals evaluate the nudge. Finally, we included four statements that reflected principles of medical ethics: autonomy (2-items, one reversed), respect to a patient, and benevolence (American Medical Association Code of Ethics).

The list of the questions and Cronbach’s α are included in Table 2.

### Data analysis

#### Comparing evaluations within each population

We used comparative statistics to examine physicians’ and non-clinicians’ evaluations of each vignette. The ratings by physicians and non-clinicians were analyzed separately with MANOVA analysis. This method allowed us to test several dependent variables accounting for multiple comparison and interrelations between the variables. The following interaction was tested 2 (patient’s initial bias: present; absent) x 2 (physician’s decision about nudge use: yes; no). We repeated the same analysis for each population separately.

#### Comparing evaluations between participants’ populations

We included the ratings by both the physicians and the non-clinicians to compare whether their evaluations were different within each vignette. To reduce the complexity of the analysis, only participants who were randomized to read the vignettes describing the physician using the nudge were included in this analysis. The following interaction 2 (patient’s initial bias: present; absent) x 2 (participants: physicians; non-clinicians) was tested with the MANOVA procedure. It is important to note that the same analysis among those who read the vignettes without the nudge revealed the patterns supporting the finding of this analysis.

## Results

### Sample

In total, 336 non-clinicians and 89 resident physicians completed the survey. The response rate among resident physicians was 56%. The participants’ demographics are in Table 3; physicians’ specialties are included in Table 4.

**Table 3:** Demographic characteristics of physicians and non-clinicians.

	Non-clinicians (n = 336)	Physicians (n = 89)
Age in years, mean (SD)	32 (10)	30 (2)
Gender: Female	64%	38%
Race: White	78%	62%

**Table 4:** Physicians specialty.

Residency	n	%
Anesthesiology	14	15.9
Dermatology	3	3.4
Emergency Medicine	10	11.4
Family Medicine & Community Health	4	4.5
General Surgery	1	1.1
Internal Medicine	31	35.2
Neurology	3	3.4
Obstetrics and Gynecology	4	4.5
Ophthalmology	2	2.3
Orthopedics	5	5.7
Otolaryngology	1	1.1
Physical Medicine and Rehabilitation	5	5.7
Psychiatry	1	1.1
Radiation Oncology	2	2.3
Urology	2	2.3
Unknown	1	1.1
Total	89	100

### Comparing evaluations within each population

#### Non-clinicians’ judgment

First, we ran the analysis for each component separately by vignette and by population. The MANOVA results indicated a non-significant main effect of the presence/absence of decision-making bias in the non-clinicians’ judgments (Vignette 1: *p* = .68; Vignette 2: *p* = .77) and the non-significant effect of the interaction between bias x nudge (Vignette 1: *p* = .07; Vignette 2: *p* = .76). However, there was a significant effect of whether nudge was present (Vignette 1: F(8, 325) = 16.10, *p* < .001; Vignette 2: F(8, 325) = 33.81, *p* < .001), suggesting that non-clinicians perceived using decision-making nudges more positively than not using them, regardless of whether the patient in the vignette demonstrated a decision-making bias. Importantly, in the vignette1 (hospice endorsement), there was no difference in patients’ evaluations of autonomy. If the nudge was present, participants evaluated patients’ autonomy the same as participants in our baseline group, which did include nudge or bias. The results for both vignettes are presented in Table 5.

**Table 5:** Non-clinicians’ evaluations of communications by domain.

	Advice for Hospice Care					Advice for Chemotherapy				
	No Nudge, *M*		Nudge, *M*		Comparison Nudge vs. No Nudge	No Nudge, *M*		Nudge, *M*		Comparison Nudge vs. No Nudge
	No Bias	Bias	No Bias	Bias	F, and p-value	No Bias	Bias	No Bias	Bias	F, and p-value
Ethics	**4.96**	4.47	5.30	5.63	27.26, *p* < .001	**4.36**	4.53	5.65	5.68	75.66, *p* < .001
Desirability	**3.94**	3.57	5.10	5.40	70.32, *p* < .001	**3.33**	3.34	5.39	5.53	161.34, *p* < .001
Acceptability	**4.88**	4.49	5.38	5.52	21.57, *p* < .001	**4.32**	4.56	5.68	5.81	65.11, *p* < .001
Effectiveness	**3.87**	3.58	4.88	4.88	72.84, *p* < .001	**3.28**	3.51	5.32	5.34	224.36, *p* < .001
*Autonomy*	**4.64**	4.37	4.58	4.66	0.52, *p* = .47	**4.77**	4.63	5.09	5.20	11.64, *p* < .001
Benevolence	**4.51**	3.94	5.46	5.67	75.09, *p* < .001	**3.62**	3.74	5.71	5.84	190.77, *p* < .001
Respect to Patient	**5.06**	4.49	5.30	5.41	17.15, *p* < .001	**4.57**	4.48	5.58	5.65	63.21, *p* < .001
Trust	**4.63**	4.41	4.99	4.84	7.20, *p* < .01	**4.25**	4.10	5.49	5.49	87.40, *p* < .001

The baseline group is bolded, in this group participants observed communication without nudge and without bias.

#### Physicians’ judgments

Second, we ran the same analysis for the physician population. Similarly, as in non-clinician population, the MANOVA test revealed a non-significant main effect of the interaction between bias and nudge (Vignette 1: *p* = .83; Vignette 2: *p* = .45). In the analysis of Vignette 1, we found a significant effect of bias (F(8, 78) = 2.15, *p* = .04). These results indicated that physicians evaluated communications as more acceptable and trustworthy if bias was absent. These relationships were not influenced by presence or absence of the nudge. This effect was not observed in the second Vignette (*p* = .73).

Like the non-clinician population, physicians evaluated the communications more positively when they used decision-making nudges than when they did not use them, (Vignette 1: F(8, 78) = 16.09, *p* < .001; Vignette 2: F(8, 78) = 7.42, *p* < .001), regardless of whether the patient in the vignette demonstrated a decision-making bias. Results for both vignettes are presented in Table 6.

**Table 6:** Physicians’ evaluations of communications by domain.

	Advice for Hospice Care					Advice for Chemotherapy				
	No Nudge, *M*		Nudge, *M*		Comparison Nudge vs. No Nudge	No Nudge, *M*		Nudge, *M*		Comparison Nudge vs. No Nudge
	No Bias	Bias	No Bias	Bias	F, and p-value	No Bias	Bias	No Bias	Bias	F, and p-value
Ethics	**4.50**	4.30	6.09	6.00	39.75, *p* < .001	**4.59**	3.96	5.23	5.50	15.63, *p* < .001
Desirability	**3.85**	2.83	5.87	5.39	54.43, *p <* .001	**3.59**	2.96	4.45	4.73	13.33, *p* < .001
Acceptability	**4.90**	4.70*	6.09	6.13*	22.95, *p* < .001	**4.86**	4.04	5.50	5.41	9.27, *p* < .01
Effectiveness	**2.98**	2.78	5.15	4.83	102.75, *p* < .001	**3.59**	2.83	4.55	4.73	31.10, *p* < .001
Autonomy	**4.28**	4.54	5.19	5.08	7.04, *p* < .01	**4.70**	4.39	4.32	4.89	0.03, *p* = .87
Benevolence	**3.80**	3.65	6.13	5.48	47.11, *p* < .001	**4.14**	3.30	5.05	5.55	28.62, *p* < .001
Respect to Patient	**4.30**	4.17	6.09	5.74	46.53, *p* < .001	**4.59**	4.13	5.32	5.27	9.77, *p* < .01
Trust	**4.95**	4.17*	5.57	5.09*	10.41, *p* < .01	**4.64**	4.26	5.27	5.32	9.50, *p* < .01

The baseline group is bolded, in this group participants observed communication without nudge and without bias.

* Patients evaluated approach as more acceptable and trustworthy when bias was not present, independently if nudge was present or absent.

### Comparing evaluations between participants’ populations

#### Vignette 1

To compare physicians’ and patients’ evaluations of nudges, we included the data from both physicians and non-clinicians. In the first analysis, we included physicians and non-clinicians who read Vignette 1 with the nudge and hospice endorsement. MANOVA analysis revealed that there was no effect of the decision-making bias on participants’ evaluations (Vignette 1: *p* = .35) and there was no effect of the interaction between bias and populations of non-clinicians/physicians (Vignette 1: *p* = .58). However, there was the main effect of the populations’ evaluations (Vignette 1: F(8, 203) = 2.16, *p* = .032), indicating that physicians evaluated nudges endorsing hospice more positively than non-clinicians, independently of whether a decision-making bias was present. Uncovering this analysis, we found that physicians evaluated nudges more favorably in 6 out of 8 domains. In two domains, benevolence and effectiveness, the ratings of the non-clinicians and the physicians did not differ. Table 7 summarizes the statics by all domains.

**Table 7:** Comparing physicians’ and patients’ evaluations of the communication, in which hospice care was endorsed.

	Hospice is endorsed: Nudge					Chemotherapy is endorsed: Nudge				
	Non-clinicians, *M*		Physicians, *M*		Non-clinicians vs. physicians	Non-clinicians, *M*		Physicians, *M*		Non-clinicians vs. physicians
	No Bias	Bias	No Bias	Bias	F, and p-value	No Bias	Bias	No Bias	Bias	F, and p-value
Ethics	5.30	5.63	6.09	6.00	10.10, *p*. = 002	5.65	5.68	5.23	5.50	2.84, *p* = .09
Desirability	5.10	5.40	5.87	5.39	2.91, *p =* .09	5.39	5.53	4.45	4.73	13.61. *p* < .001
Acceptability	5.38	5.52	6.09	6.13	8.52, *p =* .004	5.68	5.81	5.50	5.41	1.52, *p* = .22
Effectiveness	4.88	4.88	5.15	4.83	0.49, *p =* .49	5.32	5.34	4.55	4.73	18.07, *p* < .001
*Autonomy*	*4*.*58*	*4*.*66*	5.20	5.09	6.44, *p* = .01	5.10	5.20	4.32	4.89	6.91, *p* < .01
Benevolence	5.46	5.67	6.13	5.48	1.43, *p =* .23	5.71	5.84	5.05	5.55	6.09, *p* = .02
Respect to Patient	5.30	5.41	6.09	5.74	8.81, *p =* .003	5.58	5.65	5.32	5.27	2.96, *p* = .09
Trust	4.99	4.84	5.57	5.09	4.37, *p =* .04	5.49	5.49	5.27	5.32	1.14, *p* = .29

At the next step, we included age and gender as covariates. MANOVA showed non-significant impact of age (*p* = .13) and gender (*p* = .14) on the dependent variables. After we included covariates the analysis showed similar results to the main analysis reported above. There was no significant main effect of a bias or bias x population interaction. However the main effect of populations remained significant (F(8, 201) = 2.99, p < .01), suggesting that physicians evaluate the nudge more positively in this situation.

#### Vignette 2

In the next analysis, we included physicians and non-clinicians who read Vignette 2 with the nudge and chemotherapy endorsement. The MANOVA analysis revealed that there was no effect of the bias on participants’ evaluations (Vignette 2: *p* = .41), and there was no effect of the interaction between bias and population of non-clinicians/physicians (Vignette 2: *p* = .59). However, there was the main effect of the populations’ evaluations (Vignette 2: F(8, 201) = 3.25, *p* = .002), indicating that non-clinicians evaluated nudges endorsing chemotherapy more positively than physicians. Non-clinicians evaluated 6 out of 8 domains more favorably than clinicians. Both populations evaluated acceptability and trust similarly. Table 7 summarizes the results by domain.

In the next analysis, we included age and gender as covariates. MANOVA showed a marginal effect of age (F(8, 199) = 1.91, *p* = .07). Uncovering, this effect we found that older individuals tended to evaluate nudges more positively. The gender covariate significantly influenced the dependent variables (F(8, 199) = 2.27, *p* = .02), suggesting that male participants evaluated nudges more positively than female participants. Notably, when we included age and gender as covariates, the analysis showed similar results to the main analysis reported above. There was no significant main effect of a bias or bias x population interaction. However the main effect of populations remained significant (F(8, 199) = 3.09, *p* < .01) suggesting that in this case, non-clinicians evaluated nudges more positively.

## Discussion

These results demonstrate that physicians and non-clinicians generally view physicians’ use of decision-making nudges to be ethical, acceptable, and desirable in end-of-life decision making. These findings add to prior evidence that stakeholders view nudges as ethical, acceptable, and desirable [13, 25] by expanding this evidence to clinical situations in which there is a less clear “best choice” for patients. Both physicians and non-clinicians evaluated nudges positively even in the absence of patients’ having a decision-making bias. In practice, key stakeholders will likely perceive nudges in complex and preference-sensitive clinical decisions to be helpful and appropriate.

Beyond this general conclusion, our research suggest several important steps that could contribute to ensuring that physicians and patients would consider behavioral nudges favorably in clinical settings.

First, while assessing the perceptions of nudges, patients’ autonomy should be evaluated as a standalone concept. We found that non-clinicians and physicians perceived autonomy of communications differently than other components of ethics. Non-clinicians in Vignette 1 and physicians in Vignette 2 rated patients’ autonomy similarly when there was or was not nudging. Nudging was not perceived as a threat to patients’ autonomy in our study. However, in other cases, nudges might be evaluated as ethical and acceptable but still be perceived as a threat to individuals’ autonomy (e.g., nudging Jehovah's Witnesses for blood transfusion). To identify such cases, it is important to include the autonomy component in future research that aims to access patients and physicians’ perceptions of nudges.

Second, before nudges are implemented, it is important to obtain the perspectives on nudging among all communicators. The comparison of views between those who influence (physicians) and those who experience the influence (non-clinicians) did reveal important differences in the degree to which each population endorsed the use of nudges as a function of the particular kind of clinical option that was being considered. Physicians supported nudges that promoted hospice care more than did non-clinicians, whereas non-clinicians supported nudges that promoted chemotherapy more than did physicians. These findings are consistent with two lines of prior research. First, physicians are more likely than patients to choose less aggressive care in the setting of advancing illness [27]. Second, individuals support nudges more when they are consistent with their views [13]. Physicians’ greater support of nudges that endorse hopsice care and patients’ greater support of nudges that endorse chemotherapy provides additional evidence that individuals tend to favor nudges if their influence is consistent with their values. Further research should explore whether physicians would consistently support nudges promoting comfort-oriented care more strongly than non-clinicians, as this may be an important limitation to the use of nudges in clinical care.

Third, decision-making bias in patients’ judgments might have little or no influence on the evaluation of nudges in communications. We expected that participants would evaluate nudges more positively if nudges counteract a decision-making bias in patients’ judgments. However, we did not find this to be the case. Providing information about patients’ biases did not influence participants’ evaluations of nudges. However, one possibility is that most participants’ assumed such bias was present, regardless of this information. Thus, inherent in the reported perception of nudges may be the role of nudges in overcoming bias. More research is needed to deepen our understanding of the theoretical relation between the inferred decision-making bias of patients and viewing favorably the nudging of patients. Perceptions of nudges might be less favorable if participants learn that patients carefully weighed the pros and cons of each option, while formulated their preferences.

This study has several limitations. First, we tested only the nudges of information framing and social comparison. Stakeholders’ views of nudges are known to vary by type, although the types of nudges we tested have been viewed as more controversial than others [20]. Second, participants evaluated vignettes sequentially, which may have influenced their ratings of the second vignette. To address this problem, we randomized aspects of each vignette independently within each participant. The difference in the vignette evaluations between the participants’ populations signals that the sequence has a minimal impact on participants’ rating. Third, this study includes hypothetical vignettes that reduces the complexity of actual decision making. Fourth, responses from the non-clinician population were collected via the Mturk on-line panel. While there is evidence to suggest that Mturk participants provide reliable behavioral data [17, 18, 28], further research should address both the third and fourth limitations by collecting data among people who are actually dealing with the decisions described in our vignettes. Fifth, “effectiveness” and “autonomy” variables consisted of two averaged items each. The alfa coefficients were relatively low, suggesting that there is potential noise in the responses. That requires additional caution in interpreting results and further research to address this limitation.

Comparing participants’ populations, we found that age and gender were significant predictors of participants’ evaluations in the second vignette. While these results did not influence our main story, further investigation is needed. It would be interesting to address the issue of why and when older participants are more likely to evaluate nudges more positively. Considering gender, it might be that female participants for the second vignette view nudges less favorably than male participants because the gender of the patients was explicitly female. It is difficult to know, but there was not some simple “similarity” effect because the gender of the patients in the first vignette was explicitly male, and there was no evidence that the male participants viewed nudges less favorably than female participants. Nonetheless, in future studies the gender of participants should be taken into account.

## Conclusion

We found that key stakeholders in medical decision making support the use of nudges by physicians to promote choices that are intended to serve patients’ best interests. Our findings expand prior research supporting the use of nudges by confirming positive views of communication nudges even in complex, end-of-life decision making. Further research should explore stakeholders’ views of nudges when applied to actual clinical settings and examine the effects of nudges on choices and patient outcomes. If acceptable and effective, nudges may be an important decision-making tool to improve the alignment of treatment choices with true patient preferences.

## Supporting information

S1 Table Experimental materials. (PDF)

S2 Table Dependent variables. (PDF)
