# Supplementary material for: Perspectives on using decision-making nudges in physician-patient communications
Source: PLoS One. 2018 Sep 19;13(9):e0202874. doi: 10.1371/journal.pone.0202874 (PMC6145510; doi:10.1371/journal.pone.0202874)
Supplement: S1 Table — (PDF) [file pone.0202874.s001.pdf]

**Scenario 1 (Physician recommends hospice care)**	**Scenario 2 (Physician recommends chemotherapy)**
A patient has lung cancer that has spread throughout his body. The patient wants chemotherapy. **Overestimating the power of chemotherapy, the patient fully believes that chemotherapy will help him live longer and even cure his cancer.**<br><br>Physician: His physician knows that chemotherapy is not curative and will do more harm than good for him. It will cause tiredness and pain and increase the risk of infection. As a result, his physician plans to recommend comfort care instead of chemotherapy.<br><br>Approach: A physician can employ a following decision-making approach in which he communicates information that a patient is not expecting to hear. For example, if the patient is focused on avoiding losing a chance for cure by choosing chemotherapy, the physician could re-focus the patient's attention. To do so, he should mention gains the patient could achieve by choosing comfort care instead of chemotherapy (i.e. more meaningful time with his family and friends). Prior research suggests that this decision-making approach will make it more likely that the patient will choose comfort-oriented care for advanced cancer<br><br>Physician: **His physician decides to use/not to use this decision-making approach to influence the patient's choice.**	A patient with cancer was told by her doctor that she has about 11 months to live. She chooses to receive care focused on comfort instead of chemotherapy. **During the consultation, she tells her physician that she did not choose chemotherapy because she is afraid of its "horrible" side effects. She explains that her friend told her that chemotherapy causes terrible pain and a burning sensation in the blood vessels.**<br><br>Physician: Her physician knows that chemotherapy would likely benefit her by prolonging her life up to 18-24 months. Thus, her physician plans to recommend chemotherapy.<br><br>Approach: A physician can employ a following decision-making approach to help the patient re-consider chemotherapy. The physician could inform the patient that a recent patient in a similar situation received the same treatment. It shrunk her cancer and allowed her to live longer than she would have been expected to live without any treatment. Additionally, medications helped manage the side effects of chemotherapy. Prior research has shown that this approach will make it more likely that the patient will choose chemotherapy<br><br>Physician: **Her physician decides to use/not to use this decision-making approach to influence the patient's choice.**
